# Formation of metal clusters in halloysite clay nanotubes

**DOI:** 10.1080/14686996.2016.1278352

**Published:** 2017-02-16

**Authors:** Vladimir A. Vinokurov, Anna V. Stavitskaya, Yaroslav A. Chudakov, Evgenii V. Ivanov, Lok Kumar Shrestha, Katsuhiko Ariga, Yusuf A. Darrat, Yuri M. Lvov

**Affiliations:** ^a^ Department of Physical and Colloid Chemistry, I. Gubkin Russian State University of Oil and Gas, Moscow, Russia; ^b^ WPI-MANA, National Institute for Materials Science, Tsukuba, Japan; ^c^ Institute for Micromanufacturing, Louisiana Tech University, Ruston, LA, USA

**Keywords:** Halloysite nanotubes, metals intercalation, core-shell, clay, 10 Engineering and Structural materials, 102 Porous/Nanoporous/Nanostructured materials, 103 Composites, 212 Surface and interfaces, 503 TEM, STEM, SEM, Characterization

## Abstract

We developed ceramic core-shell materials based on abundant halloysite clay nanotubes with enhanced heavy metal ions loading through Schiff base binding. These clay tubes are formed by rolling alumosilicate sheets and have diameter of *c*.50 nm, a lumen of 15 nm and length ~1 μm. This allowed for synthesis of metal nanoparticles at the selected position: (1) on the outer surface seeding 3–5 nm metal particles on the tubes; (2) inside the tube’s central lumen resulting in 10–12 nm diameter metal cores shelled with ceramic wall; and (3) smaller metal nanoparticles intercalated in the tube’s wall allowing up to 9 wt% of Ru, and Ag loading. These composite materials have high surface area providing a good support for catalytic nanoparticles, and can also be used for sorption of metal ions from aqueous solutions.

## Graphical abstract

**Figure:**
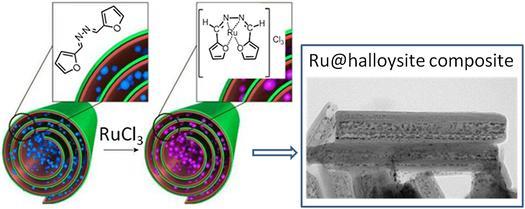

## 1. Introduction

Architectural metal nanostructures allowing for fine-tuning particle size, shape and location were based on a one-step reduction of metal salts bound to an interface of block co-polymer dendrites and amphiphile ensembles. This study used 3–5 nm diameter Pt, Au, Pd, and their bi-metallic composites to form highly porous dendritic mesostructures [1,2]. Extending this strategy, we synthesized Ru nanoparticles inside clay nanotubes.

**Figure 1.**
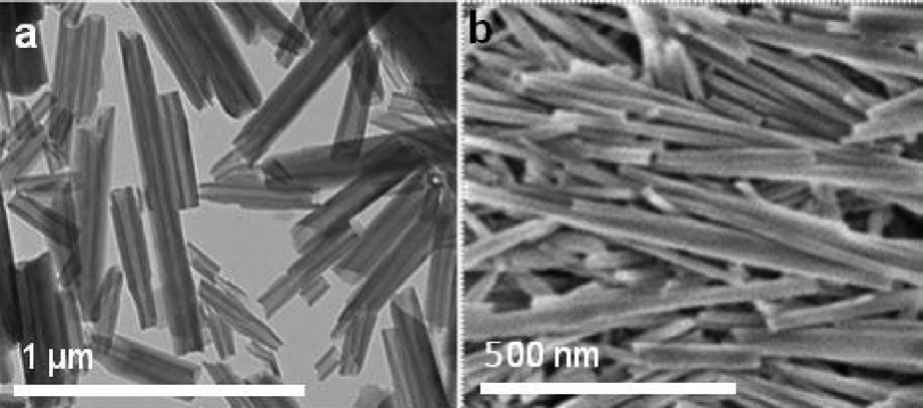
TEM (a) and SEM (b) images of halloysite nanotubes.

Halloysite nanotubes are formed by 10–15 revolutions of kaolin alumosilicate sheets and have diameters of 50–60 nm, lumens in the range of 12–15 nm, and lengths within the range of 500–900 nm (Figure 1). It is an environmentally friendly, natural, and cheap tubule nanomaterial available in large quantities (thousands of tons). Halloysite surface is composed of SiO_2_, and the tube’s interior is composed of Al_2_O_3,_ which are oppositely (negative/positive) charged in the pH range of 3–9. Halloysite may be considered as an efficient, divalent, nano-adsorbent both for cations and anions [3–7]. Adsorption of metal ions on the halloysite was used for synthesis of Fe, Co, Ni, Pd, and Ag nanoparticles on the tube surface [3,5–10]. TiO_2_ nanoparticles were bound to the nanotube surface though silane linkage [11]. It looks even more interesting to design metal–ceramic core-shell composites where metal nanoparticles placed inside the tubes will be protected against aggregation. The first core-shell halloysite system consisting of silver nanorods and Cu-Ni nanoparticles was manufactured. This system boosted the original exhaust gas catalytic efficiency at 400°C by a factor of 50 [12]. However, the metal loading efficiency and the composite production yields were rather low [9,11].

**Figure 2.**
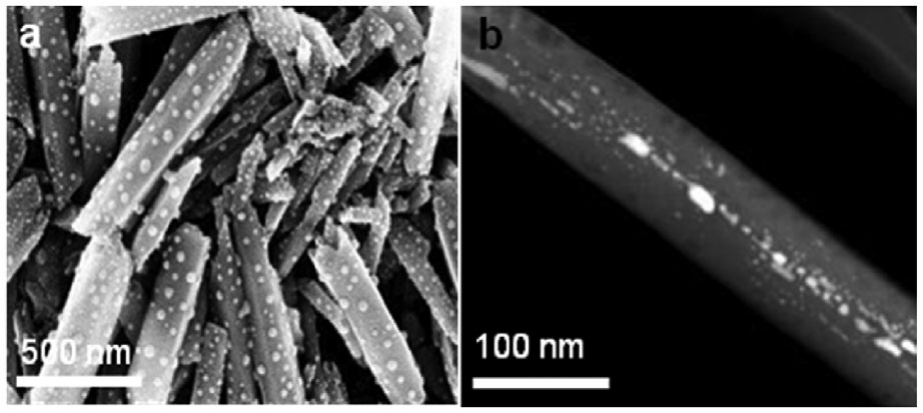
Clay nanotubes with silver particles formed at the outermost and in the inner tube surface; (a) SEM image obtained after bulk reaction of 0.5 mg ml^–1^ silver acetate mixed with halloysite; and (b) TEM image from halloysite loaded with silver acetate, washed and heated to 300 °C.

**Figure 3.**
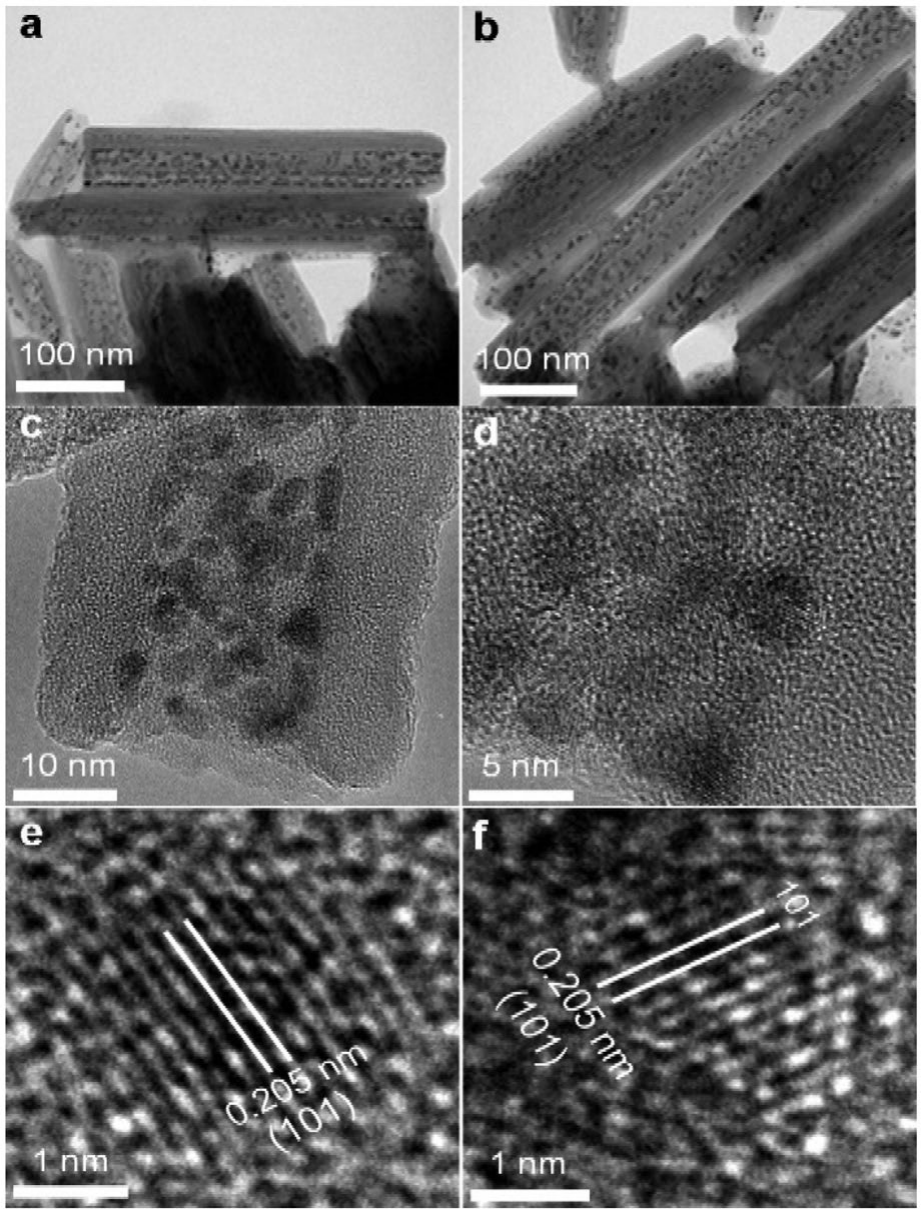
Transmission electron microscopy (TEM) of halloysite nanotubes intercalated with Ru nanoparticles bound through Schiff bases. (a-d) Morphology of the sample, (c-d) Ru nanoparticles distribution inside halloysite lumen, (e-f) HRTEM images of hcp structure of Ru nanoparticle.

Here, we suggest using clay nanotubes as a template for confined metal cluster formation. First, carrying out metal ion reduction reactions for particle formation in halloysite dispersions allows their ‘seeding’ on the tube’s outer surface. Second, metal ions are loaded selectively into 15-nm diameter halloysite lumens and nanorods or ‘peapods’ of metals are synthesized, as we have demonstrated for silver. The third step is an efficient nanoconfined metal synthesis via intercalation between the multilayer wall of the tubes and within the pocket tube defects. However, there is no simple means of direct heavy metal ion penetration into interlayer space of the alumosilicate roll. A potential intercalation can be confirmed with X-ray analysis by monitoring the 0.72-nm multilayer wall packing reflection. Halloysite tubes have a surface area of ca. 60 m^2^ g^–1^, but enabling access to the interlayer space increases its adsorption capacity to 600–700 m^2^ g^–1^, thus providing a larger space for formation of metal particles for efficient catalysis [3,4].

Some small polar organic molecules such as urea, ethylene glycol, acetonitrile, and dimethyl sulfoxide intercalate the tube wall interlayers [4]. We found that furfuraldehyde shows good intercalation abilities allowing for further formation of organic ligands for metal ions inside the nanotubes. This procedure of halloysite modification with furfuraldehyde based Schiff bases significantly enhances an intercalative loading of metal ions (Ru, Ag as well as Rh, Pt, Co, Fe) both into the lumen and also into interlayer space of the nanotubes. A simple method of metal ion inclusion from solutions based on ligand-functionalized halloysite nanotubes (ligand-HNTs) is also suggested. Subsequent reduction reactions resulted in formation of metal particles in the tube’s interior.

Further freeing the ligands allowed for repetition of the nanoparticle formation process, resulting in efficient adsorption of metal ions. Elongated heavy metal particles of 3–4 nm diameter were formed both on the inner lumen surface and in the interlayer slit-like pockets of the halloysite walls.

## 2. Experimental details

### 2.1. Materials and equipment

Halloysite clay was supplied by Applied Minerals Inc. (New York City, NY, USA). Furfuraldehyde, silver acetate, RuCl_3_ and other chemicals were purchased from Sigma-Aldrich (USA). A field emission scanning electron microscope, (SEM; Hitachi, Japan) and a transmission electron microscope (TEM; JEM-2100, JEOL, Japan) were used for imaging metal-ceramic core-shell structures. An X-ray diffractometer (Bruker-D8, Karlsruhe, Germany) was used to analyze the halloysite multilayer wall packing spacing. Local elemental analysis was carried out with energy dispersive X-ray analysis (JEM-2100, JEOL, Japan). Halloysite surface potential in aqueous dispersion was measured with a zeta potential analyzer (ZetaPlus, Brookhaven Instruments Corp., USA). Furfuraldehyde loading efficiency was estimated with thermogravimetrical analysis (TGA Q50, TA Instruments, USA) under a nitrogen flow of 25 cm^3^ min^–1^ for the sample and 10 cm^3^ min^–1^ for the balance. The explored temperature interval ranged between 25 and 900°C and the heating rate was 10°C min^–1^.

### 2.2. Synthesis of silver nanoparticles

Halloysite (5 mg) and silver acetate (10 mg) were added into deionized water (10 ml) and kept for 30 min. Then halloysite was separated by centrifugation, dried at 80°C for 3 h, and heated at 300°C for 1.5 h to decompose silver acetate into silver. For external silver particle formation, the reaction was performed in silver acetate solutions with dispersed halloysite. For the internal tube lumen synthesis, halloysite was loaded with silver acetate, then clay outermost was washed, and reaction was performed with loaded reagents.

### 2.3. Preparations of halloysite loaded with organic ligand (Schiff-base)

**Scheme 1.**
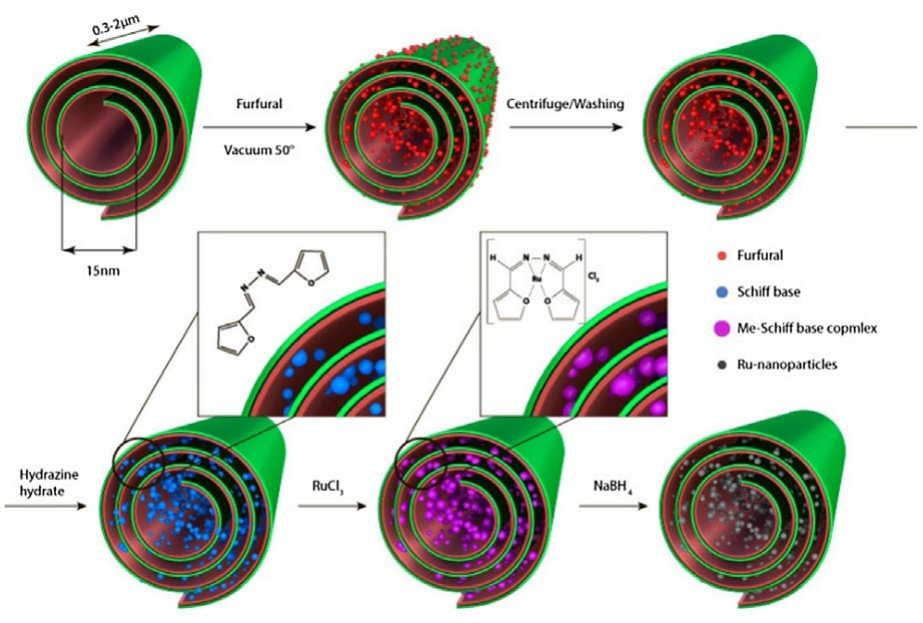
Formation of Schiff base from furfuraldehyde and hydrazine hydrate and proposed structure of the Ru-complex.

Halloysite (1 g) was mixed with furfuraldehyde (20 ml); the suspension was dispersed ultrasonically for 30 min and centrifuged to remove the chemical excess. Then hydrazine hydrate (20 ml) was added to the furfuraldehyde loaded halloysite and the mixture was stirred for 30 min under 70°C to form Schiff bases (Scheme 1). The modified halloysite underwent extensive washing/centrifugation with ethanol to remove Schiff bases from the halloysite nanotubes’ outer surfaces.

### 2.4. Synthesis of intercalated Ru-nanoparticles

Halloysite with Schiff base molecules (1 g) was dispersed ultrasonically in RuCl_3_ ethanol solution (0.05 M) to obtain a homogenous suspension and refluxed for 30 min under stirring to form an Ru Schiff base complex. The nanotubes were repeatedly washed/centrifuged to remove excess RuCl_3_. Then NaBH_4_ was added for the Ru^3+^ reduction reaction. After the reaction and degassing were completed, the modified halloysite was centrifuged and washed with water. We demonstrated three approaches in selective nanoconfined metal synthesis: (1) on the tube outer-surface; (2) in the tube central lumen; and (3) clusters bound through Schiff base reaction for formation of metal particles in the slit-like gap defects within the tube multilayer walls. This is the most interesting strategy because the area of potential metal adsorption is enhanced by a factor of 10.

### 2.5. Direct adsorption of silver acetate onto (1) and into (2) the nanotubes

(1) Performing metal ion reduction in the aqueous bulk halloysite dispersion results in nanoparticle formation, most of which are attached to the tubes’ outermost surfaces (Figure 2). Depending on the concentration of used silver acetate, we were getting larger 6–8 nm diameter silver nanoparticles or smaller 3–5 nm nanoparticles. In this approach, we did not use tube-confined synthesis and similar results may be obtained, for example, on platy montmorillonite clay [15]. However, an advantage in industrial applications of using our tubule mesoporous composites seeded with metal nanoparticles may be in the simplicity of halloysite processing which does not need exfoliation (contrary to platy montmorillonite or kaolin clays), and thus has large surface area without any additional material treatment.
(2) A more interesting approach is synthesis of metal nanoparticles inside the clay tubes, forming core-shell metal–ceramic structures. In this method, we loaded a concentrated metal reagent into the lumen of clay nanotubes, washed reagent attached outside, and then performed the reduction reaction. A high concentration of the reagent allowed complete filling of the lumens with metal. For aqueous silver acetate providing good loading of clay lumens, solid nanorods of 10–12 nm diameters were obtained [9]. However, the yield of the nanotube loaded with central nanorods was only of 4–5%. In some tubes, small silver nanoparticles were additionally formed in the elongated defects in the tube wall packing, as shown in Figure 2(b). Energy dispersive X-ray analysis proved silver formation on the nanotube samples (Supplementary Figure S1). Probably, silver acetate stayed in these narrow 3–5 nm slits as well as in point defects, more so than in the central 15-nm wide tube lumen from where the reagent may leak. To increase metal loading efficiency in the halloysite interior, we developed a technique for specific binding of ions followed by metal cluster formation through thermo-induced reduction. With this we drastically improved metal loading into the core-shell structures and the reaction product yield.

### 2.6. Organic linkage assisted metal clusters formation

To enhance metal loading into the clay nanotubes, formation of an organic linkage was employed. First, clay nanotubes were loaded with furfuraldehyde. A high furfuraldehyde loading of 5 wt% was shown with TGA analysis data but zeta potential was not changed (–32 mV). These small organic molecules were then transformed into tetradentate ligand (Schiff base) capable of binding metal cations through reaction with hydrazine hydrate. The reaction scheme is shown in Scheme 1. The tetradentate Schiff base is devoid of any ionizable protons and serves as neutral ligand forming cationic complexes [14]. Proposed structure of the Ru-complex is also shown in the scheme.

Differential scanning calorimetric (DSC) analysis proved the formation of the Schiff base inside the nanotubes (see Supplementary data, Figure S2). Characteristic phase transition at 115°C for pure Schiff base is similar to its complex with halloysite.

Such modified clay nanotubes were exposed to a solution of RuCl_3_ for 30 min. The mixture was then heated to 80°C for formation of the metal complex and treated with NaBH_4_ for metal ion reduction. The TEM images show a dense tube intercalation with Ru nanoparticles (Figure 3). These nanoparticles coated internal voids of the tubes including the larger central lumen, as well as multiple slit-like defects formed from folds in the tube wall multilayers. Contrary to the first two approaches, these nanoparticles have an elongated shape of 3–4 nm cross-section diameter and are oriented parallel to the tube axis.

Location of the metal nanoparticles along slit-like defects in the tube multilayer wall is in accordance with TEM imaging of slit-like pockets in the halloysite tubes. These folding packing defects appeared during halloysite drying accompanied with collapse of multilayer spacing from 1.0 to 0.7 nm which separate the monolithic hydrated wall to dehydrated blocks with slits [13]. Interestingly, the position of X-ray diffraction reflections corresponding to 0.72 nm spacing in the tube walls did not change after Ru clusters intercalation indicating that the nanoparticles are located only in the wall slit-voids. Similar features can be seen in Figure 2(c) with regard to silver nanoparticle distribution in the tube’s wall, but their density is much lower because no furfuraldehyde binders were used in this case.

From high resolution TEM images we obtained the Ru lattice spacing of 0.205 ± 0.05 nm corresponding to the (101) plane of the hexagonal close packed (hcp) structure. This is similar to the crystal structure observed with 3 nm diameter Ru particles [16] and corresponds to the Ru-bulk lattice. The 2–4 nm hcp Ru crystals generated a higher CO oxidation activity in comparison with face-centered cubic Ru nanoparticles, but for larger crystals catalytic efficiency decrease was observed [16,17]. It is remarkable that the yield of metal-modified tubes was very high as well as metal loading (Table 1). This demonstrates a great advantage of this ligand-based method as compared with spontaneous loading of metal complexes into the tubes’ lumens. Results of the energy dispersive X-ray analysis of the investigated sites of the samples in Figure 3(a) are summarized in Table 1 and shown in Supplementary Figure S1(b). The measured Ru percentage depended on the selected spot on the sample and varied from 6 to 10 wt%. The strong Cu K signal originated from the copper sample support.

**Table 1.** Results of elemental analysis of Ru-halloysite core-shell structures shown in Figure 3(b). Total mass of 100% does not include copper which is not part of the nanotubes.

Element	Energy, keV	Counts, ± 10	Mass, wt%
			
O K	0.525	53,544	40.6
Al K	1.486	39,007	13.8
Si K	1.739	47,374	33.7
Fe K	6.398	1505	1.2
Cu K	8.04	23,157	–
Ru L	2.558	6592	10.7
Total			100

## 3. Conclusions

We demonstrated spontaneous loading of silver ions into the halloysite nanotube lumens; however, the internal tube loading efficiency with metals was low. To enhance inner-wall and lumen metal cluster formation, we intercalated the tube with furfuraldehyde and then converted it to tetradentate ligands which have shown specific binding to Ru^3+^ ions from ethanol solution at elevated temperatures. Therefore, a simple method of metal ions exclusion from solutions based on ligand-functionalized halloysite nanotubes (ligand-HNTs) was suggested. The reduction of the sample with NaBH_4_ and cooling the halloysite composite resulted in formation of metal particles in the nanotube interior and freeing the ligands which allowed for repetition of the process and further enrichment of the tubes with metal up to 9 wt%. Metal particles of 2–5 nm diameters were formed both in the central lumen and in the interlayer spaces of the tube walls. Similar binding results were obtained for other heavy metal ions (Ag, Rh, Pt, and Co). Halloysite, our nanotemplate of choice, is inexpensive and available in large quantities (thousands of tons) in the form of clay. It is a safe, green nanomaterial [3,18,19] promising easy scale-up capabilities for industrial applications as a mesoporous carrier for metal nanocatalysts.

## Disclosure statement

No potential conflict of interest was reported by authors.

## Funding

This work was supported by Russian Science Foundation [grant number #14-19-01045 ] and US Department of Energy [grant number DE-SC0012432].

## Supplemental data

The supplemental material for this paper is available online at http://dx.doi.org/10.1080/14686996.2016.1278352

## Supplementary Material

Metal_Cluster_Formation_Supplementary_materials.docx

## References

[CIT0001] Wang L, Yamauchi Y (2010). Autoprogrammed Synthesis of Triple-Layered Au@Pd@Pt Core−Shell Nanoparticles Consisting of a Au@Pd Bimetallic Core and Nanoporous Pt Shell. J. Amer. Chem. Soc.

[CIT0002] Wang L, Yamauchi Y (2013). Metallic Nanocages: Synthesis of Bimetallic Pt–Pd Hollow Nanoparticles with Dendritic Shells by Selective Chemical Etching. J. Amer. Chem. Soc.

[CIT0003] Lvov Y, Wang W, Zhang L (2016). Halloysite Clay Nanotubes for Loading and Sustained Release of Functional Compounds. Adv. Mater.

[CIT0004] Joussein E, Petit S, Churchman J (2005). Restricted access halloysite clay minerals — a review. Clay Miner.

[CIT0005] Fu Y, Zhang L (2005). Simultaneous deposition of Ni nanoparticles and wires on a tubular halloysite template: A novel metallized ceramic microstructure. J. Sol. State Chem.

[CIT0006] Liu P, Zhao M (2009). Silver nanoparticle supported on halloysite nanotubes catalyzed reduction of 4-nitrophenol (4-NP). Appl. Surf. Sc.

[CIT0007] Zhang J, Zhang Y, Chen Y (2012). Preparation and Characterization of Novel Polyethersulfone Hybrid Ultrafiltration Membranes Bending with Modified Halloysite Nanotubes Loaded with Silver Nanoparticles. ACS Industr. Eng. Chem. Res.

[CIT0008] Zhang Y, He X, Ouyang J (2013). Palladium nanoparticles deposited on silanized halloysite nanotubes: synthesis, characterization and enhanced catalytic property. Sci. Rep.

[CIT0009] Abdullayev E, Sakakibara K, Okamoto K (2011). Natural Tubule Clay Template Synthesis of Silver Nanorods for Antibacterial Composite Coating. ACS Appl. Mater. Int.

[CIT0010] Arapov K, Lvov Y (2011). Clay tube as a template for nanoconfined synthesis. Polym. Mat. Sc. Engin.

[CIT0011] Wang R, Jiang G, Ding Y (2011). Photocatalytic activity of heterostructures based on TiO_2_ and halloysite nanotubes. Applied Mater. Interf.

[CIT0012] Sanchez-Ballester N, Ramesh G, Tanabe T (2015). Activated interiors of clay nanotubes for agglomeration-tolerant automotive exhaust remediation. J. Mater. Chem.

[CIT0013] Praus P, Turicová M, Karlíková M (2013). Nanocomposite of montmorillonite and silver nanoparticles: Characterization and application in catalytic reduction of 4-nitrophenol. Mat. Chem. Phys.

[CIT0014] Bhattacherjee CR, Goswami P, Neogi S, Dhibar S (2010). Transition metal complexes of a neutral [N_2_O_2_] donor Schiff base derived from furfuraldehyde and hydrazine hydrate: Synthesis, characterization and redox behavior. Assam Univer. J. Sci. Tech.: Phys. Sc..

[CIT0015] Kogure T (2016). Nanosized tubular clay minerals - halloysite and imogulate.

[CIT0016] Song C, Sakata O, Kumara L (2016). Size dependence of structural parameters in fcc and hcp Ru nanoparticles, revealed by Rietveld refinement analysis of high-energy X-ray diffraction data. Sci. Rep.

[CIT0017] Ye H, Wang Q, Catalano M (2016). Ru Nanoframes with an fcc Structure and enhanced catalytic properties. Nano Lett.

[CIT0018] Fakhrullina G, Akhatova F, Lvov Y (2015). Toxicity of halloysite clay nanotubes *in vivo*: a Caenorhabditis elegans study. Environ. Sci. Nano.

[CIT0019] Kryuchkova M, Danilushkina Anna, Lvov Y (2016). Evaluation of toxicity of nanoclays and graphene oxide *in vivo*: a *Paramecium caudatum* study. Environ. Sci. Nano.

