# Supplementary material for: Formation of metal clusters in halloysite clay nanotubes
Source: Sci Technol Adv Mater. 2017 Feb 16;18(1):147–51. doi: 10.1080/14686996.2016.1278352 (PMC5402758; doi:10.1080/14686996.2016.1278352)
Supplement: Metal_Cluster_Formation_Supplementary_materials.docx [file tsta_a_1278352_sm9805.docx]

**Supplementary Materials**


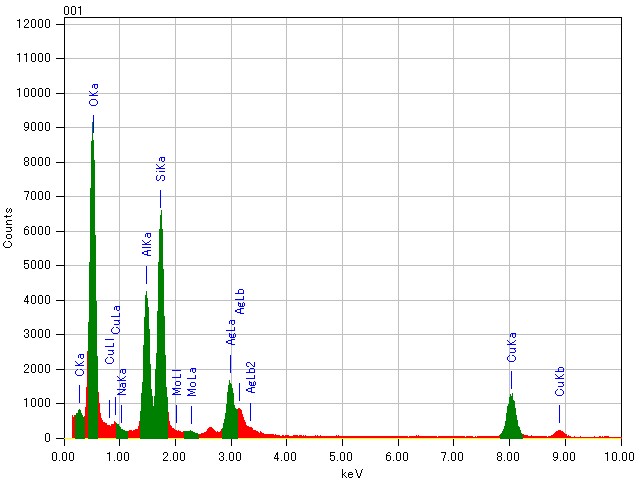

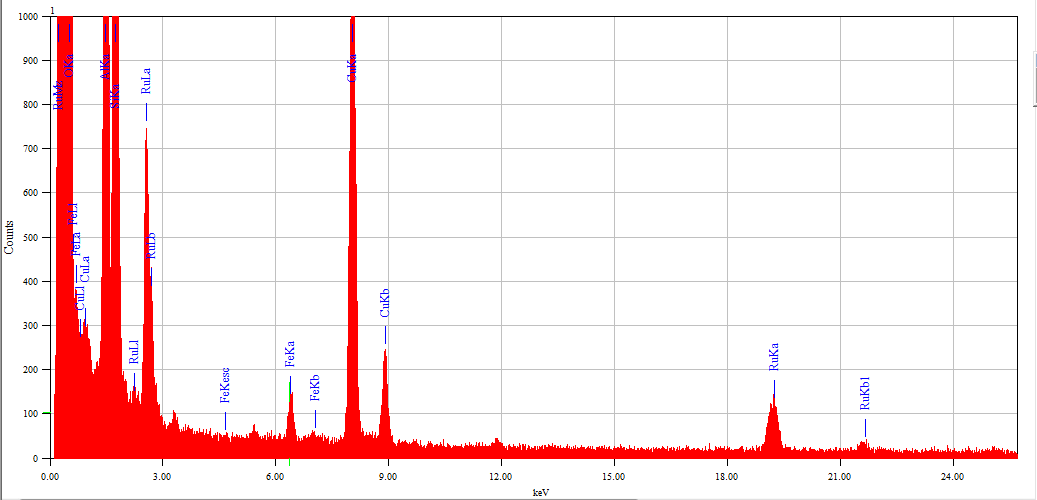


**a b**

**Fig. 1** EDS of halloysite tubule clay with synthesized Ag (a), Ru (b) nanoparticles.

**Fig.2.** Differential Scanning Calorimetry  (DSC) analysis of pristine halloysite, Schiff base and ligand-functionalized halloysite nanotubes. DSC experiments were performed with Perkin Elmer DSC 8500, heating 30 to 500 ^o^C at the rate of 10 deg / min.
